# Hypoglycemia and Hyperglycemia According to Type of Diabetes: Observations During Fully Closed-Loop Insulin Delivery in Adults With Type 1 and Type 2 Diabetes

**DOI:** 10.1177/19322968241242803

**Published:** 2024-04-13

**Authors:** Nithya Kadiyala, Malgorzata E. Wilinska, Aideen B. Daly, Munachiso Nwokolo, Rama Lakshman, Sara Hartnell, Julia Ware, Janet M. Allen, Alina Cezar, Mark L. Evans, Roman Hovorka, Charlotte K. Boughton

**Affiliations:** 1 Wellcome-MRC Institute of Metabolic Science, University of Cambridge and Addenbrooke’s Hospital, Cambridge, UK; 2 Wolfsson Diabetes and Endocrine Clinic, Cambridge University Hospitals, NHS Foundation Trust, Cambridge, UK; 3 Department of Paediatrics, University of Cambridge, Cambridge, UK

**Keywords:** artificial pancreas, fully closed-loop insulin delivery, insulin pump therapy, type 1 diabetes, type 2 diabetes, randomized trial

## Abstract

**Background:**

CamAPS HX fully closed-loop (FCL) system, with no user input required at mealtimes, has been shown to be safe and effective in adults with type 1 and type 2 diabetes. We assessed whether time spent in hypoglycemia and hyperglycemia during FCL insulin delivery in adults varied by type of diabetes over the 24-hour period.

**Methods:**

We retrospectively analyzed eight weeks of data from 52 participants (adults with type 1 diabetes and adults with insulin-treated type 2 diabetes) recruited to two single-center randomized controlled studies using FCL insulin delivery during unrestricted-living conditions. Key outcomes were time spent in hypoglycemia <70 mg/dL and marked hyperglycemia >300 mg/dL by type of diabetes.

**Results:**

The median percentage of time spent in hypoglycemia <70 mg/dL over the 24-hour period was lower for those with type 2 diabetes than for those with type 1 diabetes (median [interquartile range (IQR)] 0.43% [0.20-0.77] vs 0.86%, [0.54-1.46]; mean difference 0.46 percentage points [95% CI 0.23-0.70]; *P* < .001). Median percentage time in marked hyperglycemia >300 mg/dL was lower for those with type 2 diabetes than for those with type 1 diabetes (median [IQR] 1.8% [0.6-3.5] vs 9.3% [6.9-11.8]; mean difference 7.8 percentage points [95% CI 5.5-10.0]; *P* < .001).

**Conclusions:**

Using the FCL system, hypoglycemia and marked hyperglycemia exposure were lower in type 2 diabetes than in type 1 diabetes.

## Introduction

CamAPS HX fully closed-loop (FCL) system includes a control algorithm that directs insulin delivery based on continuous glucose monitoring (CGM) data. In contrast to hybrid closed-loop systems, the FCL system has no requirement for user input at mealtimes and reduces the burden of managing diabetes.^1^ The FCL system has been shown to improve glycemic control for adults with type 1 diabetes and suboptimal glycemic control and adults with type 2 diabetes compared to standard insulin therapy, while also being safe with no increased time spent in hypoglycemia.^1,2^

Hypoglycemia and hyperglycemia contribute to the development and progression of diabetes-related complications.^3^ Consequently, CGM-derived metrics can be useful predictors of long-term outcomes in diabetes,^4^ and analysis of these metrics can identify selective treatment targets related to hypoglycemia and hyperglycemia.^5^

In the present analysis, we investigate the burden of hypoglycemia and hyperglycemia during FCL insulin delivery in adults by type of diabetes (type 1 and type 2 diabetes) over the 24-hour period.

## Methods

We retrospectively analyzed eight weeks of data from two single-center studies using FCL insulin delivery in adults with type 1 diabetes and suboptimal glycemic control and type 2 diabetes, respectively, in unrestricted living conditions (trial registrations: NCT04977908, NCT04701424).^1,2^ Eligible adults were recruited from diabetes outpatient clinics at Addenbrooke’s Hospital (Cambridge, UK) as well as a primary care participant identification center (Granta Medical Practices, Cambridge, UK) for the study on type 2 diabetes.

Key inclusion criteria included age 18 years or older, and those with type 1 diabetes were required to be using pump therapy and have an HbA1c ≥64 mmol/mol (8.0%), while those with type 2 diabetes required treatment with subcutaneous insulin and an HbA1c ≤97 mmol/mol (11.0%). Key exclusion criteria included pregnancy, planned pregnancy or breastfeeding, severe visual or hearing impairment, and any physical or psychological disease or use of medications likely to interfere with the conduct of the trial. Participants used the FCL system for eight weeks duration in both studies. Ultra-rapid insulin Lispro (Lyumjev; Eli Lilly, Indianapolis, IN, USA) was used for the study on type 1 diabetes, and faster insulin aspart (Fiasp; Novo Nordisk, Bagsvaerd, Denmark) was used for the study on type 2 diabetes. Participants in the type 2 diabetes study continued their non-insulin diabetes therapies during the FCL period. In both studies, independent ethics approval was received, and participants gave written informed consent.

### Closed-Loop Insulin Delivery

The closed-loop app (CamAPS HX; CamDiab, Cambridge, United Kingdom) comprises the Cambridge adaptive model predictive control algorithm running on an unlocked Android smartphone. The closed-loop app receives data from the CGM device (Dexcom G6; Dexcom, CA, USA) and directs insulin delivery via an insulin pump (Dana Diabecare, SOOIL Development, Seoul, South Korea). The closed-loop app is initiated using participants’ total daily insulin dose and body weight. Every 8 to 12 minutes, in response to the sensor glucose data, the algorithm calculates an insulin infusion rate and communicates this wirelessly using Bluetooth to the insulin pump. Over time, the algorithm adapts to observed glucose patterns, enabling it to tailor insulin delivery more accurately to minimize glucose excursions.

The default target glucose used by the closed-loop algorithm is 104 mg/dL and can be adjusted as required between 80 and 200 mg/dL over different times of day and night.^1,2^ Low- and high-glucose alarms can be adjusted according to participant’s preferences. Participants were shown how to use “boost” and “ease-off” functions within the app to increase or decrease algorithm-driven insulin delivery, as required.

### Data Analysis and Statistical Methods

Key outcomes were time spent in hypoglycemia (sensor glucose <70 mg/dL) and marked hyperglycemia (sensor glucose >300 mg/dL by type of diabetes over the 24-hour period). Other outcomes included time below 54 mg/dL, time above 180 mg/dL, and time in range 70-180 mg/dL according to type of diabetes over the 24-hour period. Twenty-six participants from each study used the FCL therapy and were included in the analysis.

Data are presented as mean ± SD for normally distributed values or medians (interquartile range [IQR]) for non-normally distributed values. Independent sample *t*-tests were used to compare normally distributed values. Winsorisation was used for highly skewed endpoints. A 95% confidence interval was reported for the difference between the groups.

Outcomes were calculated using GStat software, version 2.3 (University of Cambridge, Cambridge, United Kingdom), and statistical analyses were performed using SPSS software (IBM SPSS Statistics 29.0). All *P*-values are two-tailed. *P*-values of less than .05 were considered statistically significant.

## Results

Eight weeks of data for 52 participants were included in the analysis. Table 1 lists the demographic characteristics of the study populations. Mean age was 41 ± 12 years and 59 ± 11 years for those with type 1 and 2 diabetes, respectively. Sixty-two percent of participants with type 1 and 27% of those with type 2 diabetes were female, and in both studies, 96% of participants were white. Duration of diabetes was 23.2 ± 11.0 years for those with type 1 diabetes, and 17.5 ± 8.2 years for those with type 2 diabetes. Those with type 2 diabetes required insulin therapy for 8.5 ± 6.9 years.

**Table 1.** Characteristics of Study Participants at Baseline by Type of Diabetes.

	Type 1 diabetes (n = 26)	Type 2 diabetes (n = 26)
Age (years)	41 (12)	59 (11)
Female sex, n/total n (%)	16/26 (62)	7/26 (27)
Ethnicity, n (%)	-	-
White	25 (96)	25 (96)
Black	0 (0)	0 (0)
Asian	1 (4)	1 (4)
Duration of diabetes (years)	23.2 (11.0)	17.5 (8.2)
HbA1c at baseline	-	-
Percent (%)	9.2 (1.1)	9.0 (1.4)
Millimoles per mole (mmol/mol)	77 (12)	75 (15)
Total daily insulin	-	-
Units/day (U/d)	46.1 (33.2, 74.2)	82.0 (54.8, 127.5)
Units/kg/day (U/kg/d)	0.61 (0.46, 0.86)	0.70 (0.54, 1.31)
BMI (kg/m^2^)	27.2 (4.8)	35.3 (8.6)

Data are given as n (%), mean (SD), or median (IQR).

Abbreviation: BMI, body mass index; IQR, interquartile range.

**Figure 1.**
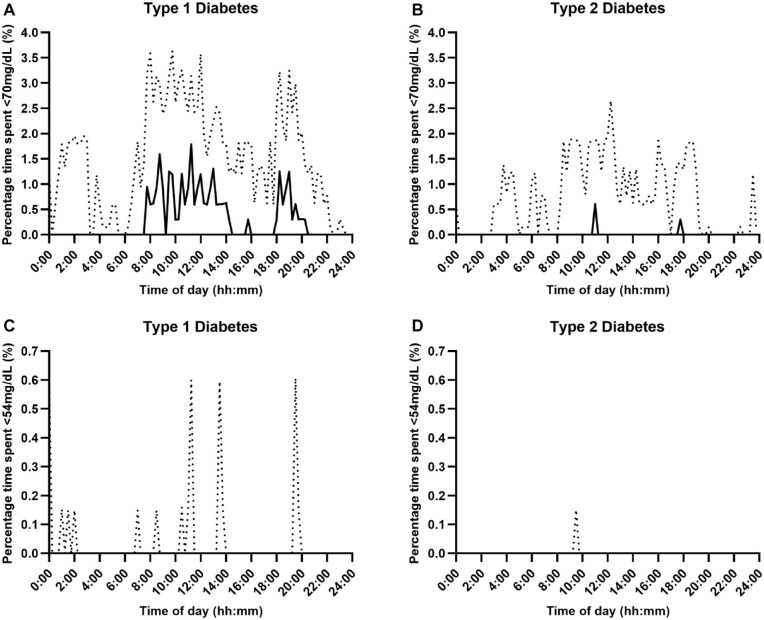
Percentage time with sensor glucose below range (Panel A and B: <70 mg/dL, Panel C and D: <54 mg/dL) over a 24-hour period using fully closed-loop therapy in adults with type 1 and type 2 diabetes (median [black line] and IQR [dotted line]).

**Figure 2.**
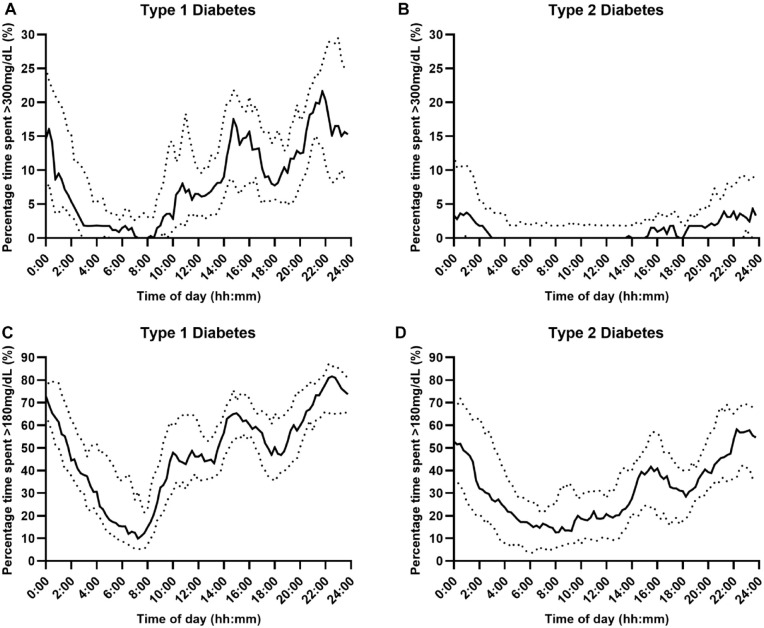
Percentage time with sensor glucose above range (Panel A and B: >300 mg/dL, Panel C and D: >180 mg/dL) over a 24-hour period using fully closed-loop therapy in adults with type 1 and type 2 diabetes (median [black line] and IQR [dotted line]).

**Figure 3.**
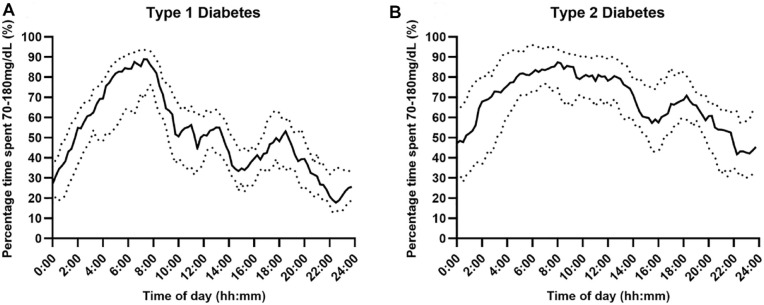
Percentage time with sensor glucose in range (Panel A and B: 70-180 mg/dL) over a 24-hour period using fully closed-loop therapy in adults with type 1 and type 2 diabetes (median [black line] and IQR [dotted line]).

Glycemic outcomes by type of diabetes are presented in Table 2. The distribution of the median time spent at sensor glucose levels, including time below range (<70 mg/dL, <54 mg/dL), time above range (>300 mg/dL, >180 mg/dL), and time in range (70-180 mg/dL), over the 24-hour period according to type of diabetes is shown in Figures 1 to 3, respectively.

**Table 2.** Glycemic and Insulin Outcomes as per Type of Diabetes.

	Type 1 diabetes (n = 26)	Type 2 diabetes (n = 26)	95% CI for treatment difference	*P* value
Percent of time with sensor glucose level (%)	-	-	-	-
<54 mg/dL	0.12 (0.03, 0.27)	0.04 (0.01, 0.08)	0.09 (0.04, 0.15)	.001
<70 mg/dL	0.86 (0.54, 1.46)	0.43 (0.20, 0.77)	0.46 (0.23, 0.70)	<.001
70-180 mg/dL	49.9 ± 9.6	66.3 ± 14.9	−16.4 (−23.4, −9.5)	<.001
>180 mg/dL	49.1 ± 9.9	33.2 ± 14.8	16.0 (9.0, 23.0)	<.001
>300 mg/dL	9.3 (6.9, 11.8)	1.8 (0.6, 3.5)	7.8 (5.5, 10.0)	<.001
Mean glucose (mg/dL)	193.2 ± 19.7	165.1 ± 22.4	28.1 (16.3, 39.8)	<.001
Glucose SD (mg/dL)	74.7 ± 10.9	53.7 ± 14.3	20.9 (13.8, 28.0)	<.001
Glucose CV (%)	38.7 ± 4.4	32.2 ± 5.7	6.5 (3.7, 9.4)	<.001

SD, standard deviation; CV, coefficient of variation.

Data are given as mean ± SD or median (IQR).

The median percentage of time spent in hypoglycemia (<70 mg/dL) over the 24-hour period was low in both populations but was lower at 0.43%, IQR 0.20-0.77, in those with type 2 diabetes than in those with type 1 diabetes (0.86%, IQR 0.54-1.46); with a mean difference of 0.46 percentage points (95% CI 0.23-0.70; *P* < .001). Hypoglycemic burden appeared to be lower overnight in both populations. For those with type 1 diabetes, median percentage time spent in hypoglycemia (<70 mg/dL) was greatest between 08:00-14:00 and 18:00–20:00 hours (Figure 1, Panel A). Median percentage time in hyperglycemia (>300 mg/dL) over the 24-hour period was lower for those with type 2 diabetes (1.8%, IQR: 0.6-3.5) than for those with type 1 diabetes (9.3%, IQR: 6.9-11.8); mean difference 7.8 percentage points (95% CI 5.5-10.0; *P* < .001). Hyperglycemic excursions occurred predominantly in the daytime and tended to rise between 14:30-16:00 and 20:30-00:30, peaking at 21:00-22:00 hours, for those with type 1 diabetes (Figure 2, Panel A), and for those with type 2, hyperglycemic excursions increased between 15:00 and 03:00, but in general, it remained low peaking at 4.3% at 23:30 (Figure 2, Panel B).

Time below sensor glucose <54 mg/dL was low in both populations but was lower for adults with type 2 diabetes (0.04%, IQR 0.01-0.08) than for those with type 1 diabetes (0.12%, IQR 0.03-0.27; mean difference 0.09 percentage points [95% CI 0.04-0.15]; *P* = .001), and for both populations, this occurred predominantly during the daytime (Figure 1, Panel C and D). For those with type 2 diabetes compared to type 1 diabetes, there was less time spent above the range >180 mg/dL (mean ± SD: 33.2% ± 14.8% vs 49.1% ± 9.9%, mean difference 16.0 percentage points [95% CI 9.0-23.0], *P* < .001), peaking between 22:00-00:00 (Figure 2, Panel C) and 21:00-23:00, respectively (Figure 2, Panel D).

Time in range (70-180 mg/dL) was higher for those with type 2 diabetes than for those with type 1 diabetes (mean ± SD 66.3 ± 14.9 vs 49.9 ± 9.6; mean difference 16.4 percentage points [95% CI 9.5-23.4], *P* < .001). Time in range was greatest during the early morning hours for both populations (05:00-09:00), with a more even distribution during the daytime for those with type 2 diabetes than for those with type 1 diabetes (Figure 3).

Other glycemic outcomes including the mean glucose and measures of glycemic variability such as the glucose SD and glucose CV were lower for those with type 2 diabetes than for those with type 1 diabetes (Table 2).

## Discussion

In this retrospective analysis of two FCL studies in adults with type 1 diabetes and insulin-treated type 2 diabetes during unrestricted-living conditions, time spent in hypoglycemia and hyperglycemia was lower for those with type 2 diabetes.

Adults with type 2 diabetes experienced less time in hypoglycemia and hyperglycemia than those with type 1 diabetes, and these differences were most pronounced during daytime. This is in line with previous findings^6^ and can be explained by the different pathophysiology of the conditions. People with type 2 diabetes typically retain some level of endogenous insulin production, which sets them apart from individuals with type 1 diabetes.^7^ This residual insulin production in type 2 diabetes likely results in less pronounced fluctuations in glucose levels, especially when external factors like food intake and physical activity come into play during the daytime. This is also reflected in the lower glucose variability measures (standard deviation and coefficient of variation) in adults with type 2 diabetes compared to adults with type 1 diabetes (Table 2).

Hypoglycemic and hyperglycemic excursions appeared to be most frequent during the daytime from 09:00 onwards, with time in range being highest during early morning (05:00-09:00). This is likely attributable to the multiple factors that can influence glucose levels during the daytime, for example, prandial glucose excursions, exercise, and stress.^6^

International guidelines recommend adults with type 1 and type 2 diabetes should spend <4% of time below target glucose range <70 mg/dL.^5^ This was achieved by all participants using the FCL system. International guidelines recommend adults should spend at least 70% of time per day in range and less than 25% time per day above range (>180 mg/dL).^5^ Using the FCL system, few participants met these guidelines although they were achieved in a larger percentage of those with type 2 diabetes than among those with type 1 diabetes (time in range [70-180 mg/dL]: 38% vs 4%, time above range [>180 mg/dL]: 31% vs 0%). This could be attributed to the high mean baseline HbA1c of the study participants (Table 1), which is a key factor in determining time in range achieved with the use of a closed-loop system.

Our results suggest that meal announcements during the daytime to decrease glycemic excursions may be important, particularly for those with type 1 diabetes, to achieve the target time in range. However, this adds significant burden for users and is not always feasible for those with lower levels of engagement.

Strengths of this analysis include the use of the same algorithm across two different types of diabetes populations that are comparable in terms of higher baseline HbA1c as well as the unrestricted living study design. The limitations of this study include the retrospective analysis, differences in age of populations, lack of ethnic diversity, and differences in study design including the insulin being used. The participants with type 1 diabetes might not be representative of the general population due to the requirement of insulin pump therapy. Baseline C-peptide and details regarding exercise and meals or the frequency of “boost” and “ease-off” use were not collected in these studies.

In conclusion, using the FCL system, time spent in hypoglycemia and hyperglycemia was lower for those with type 2 diabetes than for those with type 1 diabetes.
